# Inorganic Polyphosphate Suppresses Lipopolysaccharide-Induced Inducible Nitric Oxide Synthase (iNOS) Expression in Macrophages

**DOI:** 10.1371/journal.pone.0074650

**Published:** 2013-09-09

**Authors:** Kana Harada, Toshikazu Shiba, Kazuya Doi, Koji Morita, Takayasu Kubo, Yusuke Makihara, Adriano Piattelli, Yasumasa Akagawa

**Affiliations:** 1 Department of Advanced Prosthodontics, Institute of Biomedical and Health Sciences, Hiroshima University, Hiroshima, Japan; 2 Regenetiss Inc., Koganei, Japan; 3 Laboratory for Polyphosphate Research, The Kitasato Institute, Kitasato Institute for Life Sciences, Kitasato University, Tokyo, Japan; 4 Department of Medical, Oral and Biotechnological Sciences, Chieti-Pescara University, Chieti, Italy; St. Jude Children's Research Hospital, United States of America

## Abstract

In response to infection, macrophages produce a series of inflammatory mediators, including nitric oxide (NO), to eliminate pathogens. The production of these molecules is tightly regulated via various mechanisms, as excessive responses are often detrimental to host tissues. Here, we report that inorganic polyphosphate [poly(P)], a linear polymer of orthophosphate ubiquitously found in mammalian cells, suppresses inducible nitric oxide synthase (iNOS) expression induced by lipopolysaccharide (LPS), a cell wall component of Gram-negative bacteria, in mouse peritoneal macrophages. Poly(P) with longer chains is more potent than those with shorter chains in suppressing LPS-induced iNOS expression. In addition, poly(P) decreased LPS-induced NO release. Moreover, poly(P) suppressed iNOS mRNA expression induced by LPS stimulation, thereby indicating that poly(P) reduces LPS-induced iNOS expression by down-regulation at the mRNA level. In contrast, poly(P) did not affect the LPS-induced release of TNF, another inflammatory mediator. Poly(P) may serve as a regulatory factor of innate immunity by modulating iNOS expression in macrophages.

## Introduction

Inorganic polyphosphate [poly(P)] is a linear polymer composed of tens to hundreds of orthophosphate (Pi) residues linked by high-energy phosphate bonds and widely found in organisms ranging from bacteria to mammals [1]. In prokaryotes and lower eukaryotes, poly(P) plays a critical role in survival by providing phosphate and energy, chelating heavy metals, buffering alkaline stress, regulating gene expression and forming channels [1]. Roles of poly(P) in mammals, including humans, have been revealed in the last decade. We previously found that poly(P) stimulates cell proliferation by stabilizing the binding of fibroblast growth factor-2 to its receptors [2] and also induces the differentiation and calcification of osteoblasts [3], [4]. In the blood coagulation system, poly(P) acts as a procoagulant mediator after release from platelet granules [5]–[9]. Moreover, it has been reported that poly(P) is involved in the function of transient receptor potential ankyrin 1 and transient receptor potential melastatin 8 [10], [11], cell apoptosis [12], activation of mammalian target of rapamycin (mTOR) [13], and energy metabolism [14]. However, despite the ubiquitous distribution of poly(P) in tissues and cells, including brain, heart, kidney, lung, liver, plasma, peripheral blood mononuclear cells, and mast cells [15]–[17], current knowledge pertaining to the functions of poly(P) is limited. This prompted us to further investigate the functions of poly(P).

Upon infection, macrophages sense pathogen-associated molecular patterns through a family of pattern-recognition receptors and activate the immune system [18], [19]. Lipopolysaccharide (LPS), which is closely associated with infectious diseases such as sepsis and periodontal diseases, is a cell wall component of Gram-negative bacteria and it is recognized by macrophages via Toll-like receptor 4 (TLR4) [18], [19]. The activation of TLR4 in macrophages results in the induction of inflammatory genes such as tumor necrosis factor, interleukins and inducible nitric oxide synthase (iNOS) [20]. These molecules are essential for host defense against infection. For instance, macrophages engulf pathogens and destroy them with nitric oxide (NO) produced by iNOS [21]. However, excessive amounts of NO are also detrimental to host tissues and cells and implicated in the pathology of various diseases. Thus, NO production by iNOS is tightly regulated at the level of transcription, translation, posttranslational modification and iNOS activity [22].

In the present study, we investigated the effect of extracellular poly(P) on iNOS expression and TNF release in mouse peritoneal macrophages. Our results demonstrate that poly(P) suppresses iNOS expression and NO release but not TNF release in LPS-activated macrophages. We observed that poly(P)-mediated suppression of iNOS expression was regulated at the level of mRNA expression.

## Materials and Methods

### Ethics statement

This study was performed in accordance with Guidelines for Animal Experimentation, Hiroshima University, and approved by the Committee of Animal Experimentation, Hiroshima University.

### Materials

Reagents were obtained from the following sources: LPS from InvivoGen (San Diego, CA, USA); fluid thioglycollate medium from Nihon Pharmaceutical Co., Ltd. (Tokyo, Japan); RPMI 1640 medium, trypan blue, TaqMan® RNA-to-C_T_™ *1-Step* Kit, and TaqMan® Gene Expression Assays from Life Technologies Co. (Carlsbad, CA, USA); fetal bovine serum (FBS) from BioWest (Nuaille, France); anti-mouse iNOS antibody from Becton, Dickinson and Company (Franklin Lakes, NJ, USA); anti-mouse β-tubulin antibody from Sigma-Aldrich Co. (St. Louis, MO, USA); ECL Western Blotting Detection Reagents from GE Healthcare UK Ltd. (Amersham, UK); Cell Counting Kit-8 from Dojindo Laboratories (Kumamoto, Japan); Griess Reagent System from Promega Co. (Madison, WI, USA); RNeasy Plus Mini Kit and QIAshredder from Qiagen (Hilden, Germany); and Mouse TNF-alpha Quantikine ELISA Kit from R&D systems (Minneapolis, MN, USA).

Poly(P) was prepared as described previously [23], [24]. The purity of poly(P) was confirmed by HPLC gel filtration chromatography. HPLC was performed on a Shodex OHpak SB-803 HQ column using 100-mM NaCl solvent at a flow rate of 1.0 mL/min and a temperature of 25°C. Almost no tripolyphosphate, pyrophosphate, or Pi contaminated poly(P)_14_, poly(P)_60_, or poly(P)_130_ (the average number of phosphate residues were 14, 60 and 130, respectively) based on chromatography chart showing the detected refractive index. In addition, we measured the Pi concentrations in poly(P)_14_, poly(P)_60_, and poly(P)_130_ by the micro phosphate method using ascorbic acid and ammonium molybdate [25], and Pi concentrations were below the detection limit in all of the poly(P) samples. In this study, sodium phosphate buffer (as Pi) was used as a control for poly(P).

All other reagents were purchased from commercial sources and were of the highest available purity.

### Cell culture

One or two male C57BL/6J mice (8–12 weeks old) were used for each preparation of peritoneal macrophages. The mice were intraperitoneally injected with 3 mL of thioglycollate medium. After 3 days, peritoneal exudate cells were harvested from the peritoneal cavities. The cells were washed twice with RPMI 1640 and suspended in RPMI 1640 supplemented with 1% FBS. Aliquots of the cell suspensions were transferred to wells of 24-well plates (5×10^5^ cells) or 60-mm diameter dishes (3×10^6^ cells) and allowed to adhere for 2 h under 5% CO_2_/95% air at 37°C. Unattached cells were removed by rinsing with RPMI 1640 and the remaining adherent cells were used as macrophages for further experiments. The purity of macrophages was determined using anti-F4/80 immunostaining and was found to be more than 95%.

### iNOS detection by Western blotting

Macrophages plated in 60-mm diameter dishes in 3 mL of RPMI 1640 containing 1% FBS were pretreated with poly(P) for 5 min and stimulated with LPS for 24 h. In some experiments, macrophages were treated with poly(P) or LPS for 24 h. The cells were then washed with phosphate-buffered saline, lysed by adding sodium dodecyl sulfate (SDS) sample buffer, and sonicated. After heating to 95°C for 5 min, the protein samples were separated by SDS-polyacrylamide gel electrophoresis and blotted onto polyvinylidene difluoride membranes. The membranes were blocked with blocking buffer containing 5% skim milk for 3 h at room temperature and incubated with primary antibody against iNOS and β-tubulin overnight at 4 °C. After washing, the membranes were further incubated with horseradish peroxidase-conjugated secondary antibody for 1 h at room temperature. The membranes were then incubated with ECL Western Blotting Detection Reagents, and immunoreactivity was detected using a luminescent image analyzer, LAS-1000plus (Fuji Photo Film, Tokyo, Japan). The band intensities were quantified using ImageJ (Rasband WS, ImageJ, U. S. National Institutes of Health, Bethesda, Maryland, USA, http://imagej.nih.gov/ij/, 1997–2012) and normalized to the levels of β-tubulin.

### Cell viability assays

Cell viability was assessed by 2-(2-methoxy-4-nitrophenyl)-3-(4-nitrophenyl)-5-(2,4-disulfophenyl)-*2H*-tetrazolium (WST-8) assays, based on the reduction of the tetrazolium salt WST-8 by dehydrogenase activities in living cells, using the Cell Counting Kit-8, according to the manufacturer’s protocol. In brief, macrophages plated in 24-well plates in 0.4 mL of RPMI 1640 containing 1% FBS were pretreated with poly(P) for 5 min and stimulated with LPS for 24 h. On the other hand, macrophages were treated with H_2_O_2_ (250 µM) for 24 h, which was used as a positive control for cell death. Subsequently, 40 µL of a mixed solution of WST-8 (5 mM) and 1-methoxy-5-methylphenazinium methylsulfate (0.2 mM) was added to each well. After incubation for 1 h, 100 µL of the medium was transferred to a well of a 96-well plate and absorbance was measured at 450 nm.

Alternatively, a trypan blue exclusion assay was performed. Macrophages plated in 24-well plates in 0.4 mL of RPMI 1640 containing 1% FBS were pretreated with poly(P) for 5 min and stimulated with LPS for 24 h. Then, the cells were incubated with 0.4% trypan blue and gently rinsed with PBS. Unstained cells were counted in randomly selected fields under bright field microscopy.

### Nitrite assays

Nitrite, a metabolite of NO, was quantified using the Griess reaction to determine the amount of NO released by the cells. Macrophages plated in 24-well plates in 0.4 mL of RPMI 1640 containing 1% FBS were pretreated with poly(P) for 5 min and stimulated with LPS for 24 h. Nitrite was assayed in 50 µL aliquots of the medium using the Griess Reagent System, according to the manufacturer’s protocol.

### Real-time RT-PCR analysis of iNOS mRNA

Macrophages plated in 60-mm diameter dishes in 3 mL of RPMI 1640 containing 1% FBS were pretreated with poly(P) for 5 min and stimulated with LPS for 6 h. Total RNA was isolated from the cells using RNeasy Plus Mini Kit and QIAshredder, according to the manufacturer’s protocol. The amount of total RNA was determined by light absorption at 260 nm and 280 nm. Real-time RT-PCR was performed using TaqMan® RNA-to-C_T_™ *1-Step* Kit and TaqMan® Gene Expression Assays, according to the manufacturer’s protocol. Assay IDs for iNOS and glyceraldehydes-3-phosphate dehydrogenase (GAPDH) were Mm00440502_m1 and Mm99999915_g1, respectively. Data were obtained and analyzed using an ABI PRISM® 7700 Sequence Detection System (Applied Biosystems, Foster City, CA, USA). The expression levels of iNOS mRNA were normalized to that of GAPDH mRNA.

### TNF assays

Macrophages plated in 24-well plates in 0.4 mL of RPMI 1640 containing 1% FBS were pretreated with poly(P) for 5 min and stimulated with LPS for 3 h or 24 h. TNF was assayed in 50 µL aliquots of the medium using Mouse TNF-alpha Quantikine ELISA Kit, according to the manufacturer’s protocol.

### Statistical analysis

Statistical analysis of the data was performed using one-way analysis of variance (ANOVA) followed by pairwise comparisons using Bonferroni correction.

## Results

### Effects of poly(P) on LPS-induced iNOS protein expression in macrophages

**Figure 1:**
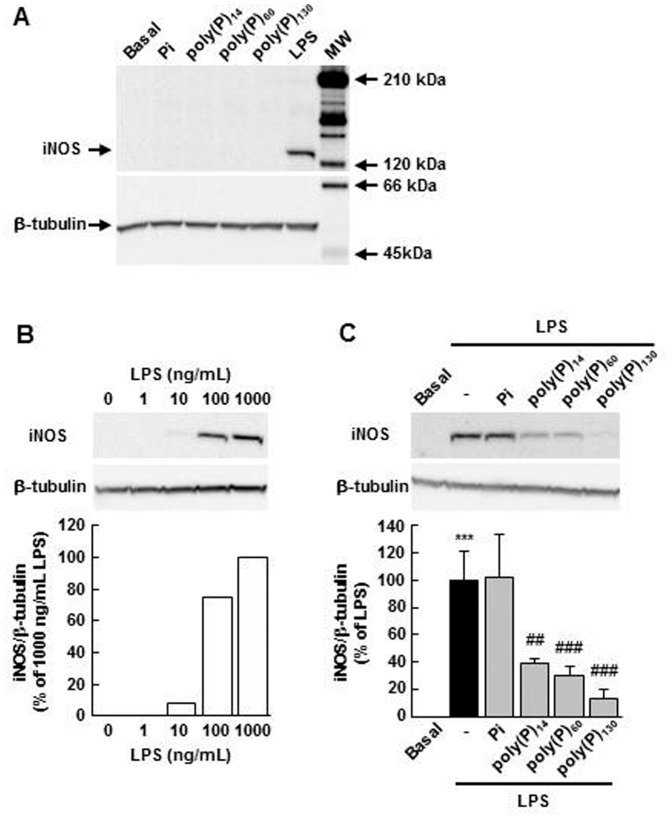
The effects of poly(P) on iNOS expression in macrophages. (A) poly(P) does not affect iNOS expression alone. The cells were treated with Pi, poly(P)_14_, poly(P)_60_, or poly(P)_130_ (1 mM) for 24 h. iNOS expression was detected by Western blotting. LPS (100 ng/mL, 24 h) was used as a positive control. A similar result was obtained in another set of independent experiments. MW, molecular weight marker. (B) Concentration dependence of LPS-induced iNOS expression. The cells were treated with the indicated concentrations of LPS for 24 h. iNOS expression was detected by Western blotting (upper) and then quantified by densitometry (lower). A similar result was obtained in another set of independent experiments. (C) poly(P) suppresses LPS-induced iNOS expression. The cells were pretreated with Pi, poly(P)_14_, poly(P)_60_, or poly(P)_130_ (1 mM) for 5 min and stimulated with LPS (100 ng/mL) for 24 h. iNOS expression was detected by Western blotting (upper) and then quantified by densitometry (lower). Values are expressed as means ± SD of the percentage of iNOS expression compared with LPS alone from three independent experiments using different cell preparations. ***p<0.001, significantly different from basal. ##*p*<0.01, ###*p*<0.001, significantly different from LPS alone (ANOVA with Bonferroni’s post-test).

We examined whether poly(P) could influence iNOS expression in macrophages using poly(P) of various chain lengths; the average number of phosphate residues were 14 [poly(P)_14_], 60 [poly(P)_60_], or 130 [poly(P)_130_]. None of these induced iNOS expression alone even at 1 mM [all poly(P) concentrations are presented in terms of phosphate residues], while a marked induction of iNOS was observed in LPS-stimulated macrophages (Figure 1A). In addition, the poly(P) degradation product Pi alone did not affect iNOS expression (Figure 1A). We next investigated whether poly(P) could modulate LPS-induced iNOS expression. In subsequent experiments, we used 100 ng/mL LPS, which induced a submaximal expression of iNOS (Figure 1B). As shown in Figure 1C, poly(P)_14_, poly(P)_60_, and poly(P)_130_ (1 mM) significantly reduced iNOS expression induced by LPS, although Pi exerted no such effect. The inhibition rates by poly(P)_14_, poly(P)_60_, and poly(P)_130_ were 61.7%±3.7%, 69.9%±6.9%, and 87.4%±6.7% (means ± SD), respectively, suggesting that poly(P) with longer chains is more potent than those with shorter chains in suppressing LPS-induced iNOS expression.

**Figure 2:**
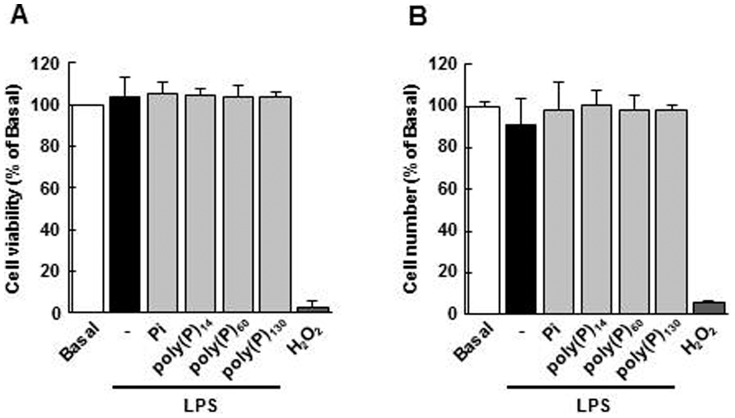
The effects of poly(P) on the viability of macrophages. The cells were pretreated with Pi, poly(P)_14_, poly(P)_60_, or poly(P)_130_ (1 mM) for 5 min and stimulated with LPS (100 ng/mL) for 24 h. Cell viability was then determined using the WST-8 assay (A) and trypan blue exclusion assay (B). H_2_O_2_ (250 µM, 24 h) was used as a positive control. Values are expressed as means ± SD of the percentage of cell viability (A) or cell number (B) compared with basal from three independent experiments using different cell preparations.

To exclude the possibility that poly(P)-mediated suppression of iNOS expression was due to the death of macrophages, we tested whether poly(P) had any effect on cell viability using the WST-8 assay and trypan blue exclusion assay. As shown in Figure 2A, poly(P)_14_, poly(P)_60_, and poly(P)_130_ as well as Pi (all at 1 mM) did not affect the viability of LPS-stimulated macrophages, whereas H_2_O_2_, a reactive oxygen species, caused cell death and decreased the formation of WST-8 formazan. Moreover, the number of trypan blue-negative cells did not change with the treatment with poly(P) (Figure 2B). These results indicate that poly(P) suppresses iNOS expression in LPS-stimulated macrophages by controlling cell function, but not cell viability.

### Concentration dependence of poly(P)-mediated suppression on LPS-induced iNOS expression

**Figure 3:**
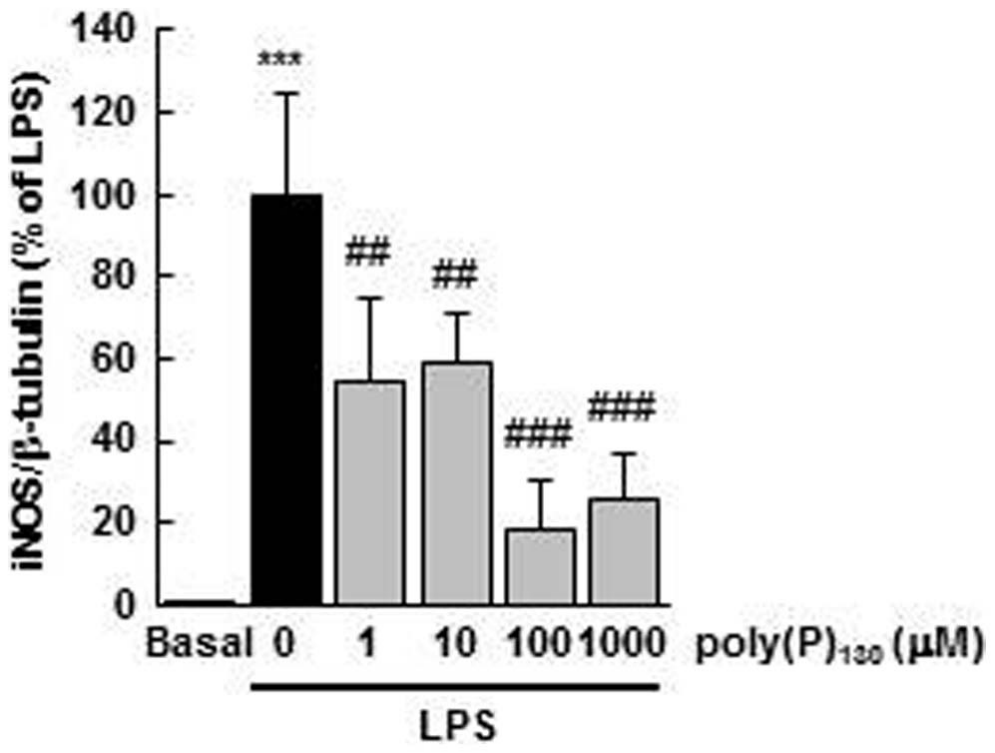
Concentration-dependent suppression of LPS-induced iNOS expression by poly(P). The cells were pretreated with the indicated concentrations of poly(P)_130_ for 5 min and stimulated with LPS (100 ng/mL) for 24 h. iNOS expression was detected by Western blotting and then quantified by densitometry. Values are expressed as means ± SD of the percentage of iNOS expression compared with LPS alone from three independent experiments using different cell preparations. ***p<0.001, significantly different from basal. ##*p*<0.01, ###*p*<0.001, significantly different from LPS alone (ANOVA with Bonferroni’s post-test).

In mammals, various tissues and cells contain micromolar concentrations of poly(P) [15], [16], [26]. Thus, we examined the concentration dependence of poly(P)-mediated suppression on LPS-induced iNOS expression. We observed that poly(P)_130_ significantly reduced LPS-induced iNOS expression even at 1 µM and exerted maximum inhibition at 100 µM (Figure 3).

### Effects of poly(P) on LPS-induced NO production

**Figure 4:**
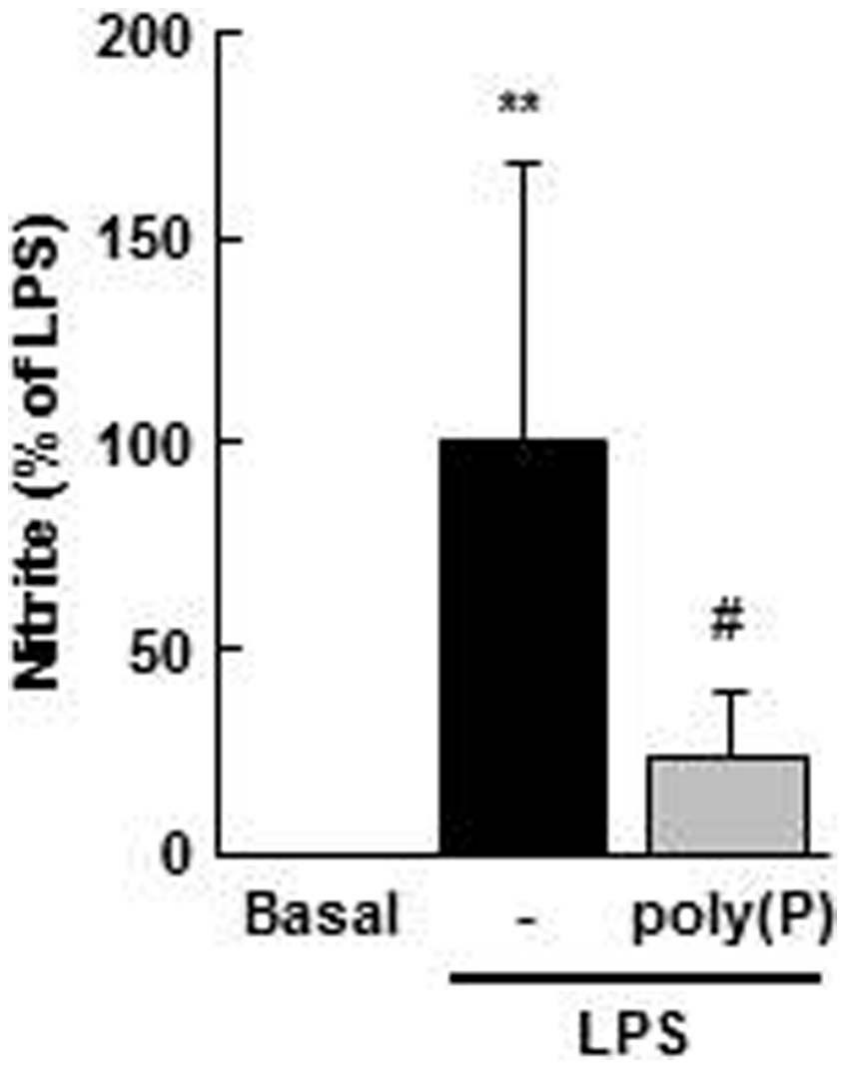
poly(P) reduces LPS-induced NO release in macrophages. The cells were pretreated with poly(P)_130_ (100 µM) for 5 min and stimulated with LPS (100 ng/mL) for 24 h. NO released (measured as nitrite) was determined by the Griess reaction. Values are expressed as means ± SD of the percentage of nitrite compared with LPS alone from six independent experiments using different cell preparations. **p<0.01, significantly different from basal. #*p*<0.05, significantly different from LPS alone (ANOVA with Bonferroni’s post-test).

iNOS produces large amounts of NO from L-arginine. The amount of NO production is primarily regulated at the expression level of iNOS, but posttranslational modification is also involved. We therefore investigated whether poly(P)-mediated suppression of iNOS expression could lead to the reduction of NO release. As shown in Figure 4, treatment of LPS-stimulated macrophages with poly(P)_130_ (100 µM) resulted in decreased NO release similar to the case of iNOS expression.

### Effects of poly(P) on LPS-induced iNOS mRNA expression

**Figure 5:**
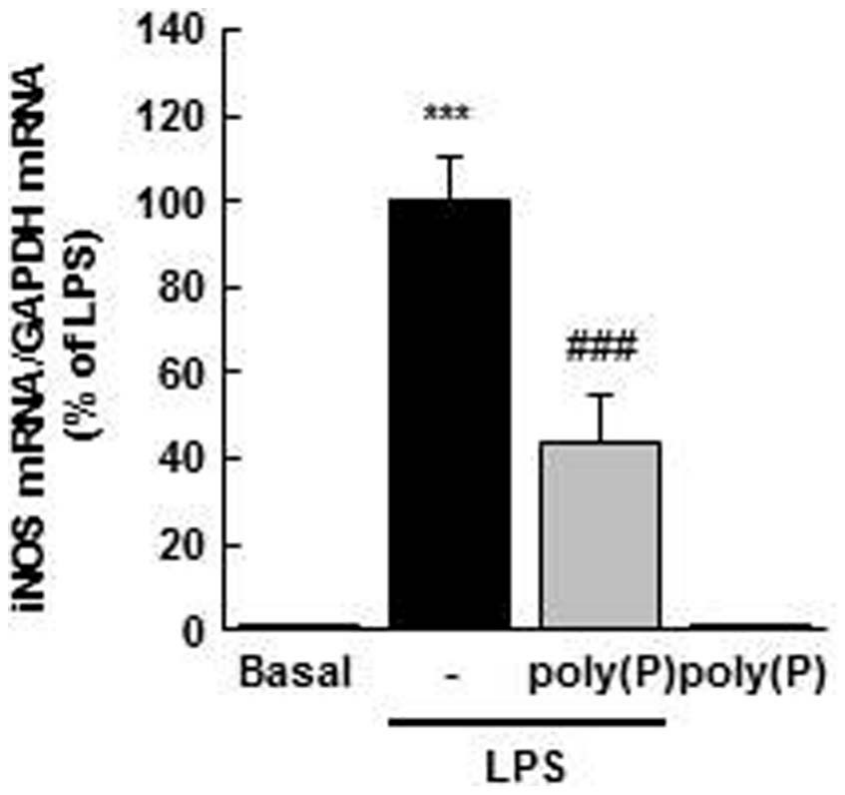
poly(P) suppresses LPS-induced iNOS mRNA expression in macrophages. The cells were pretreated with poly(P)_130_ (100 µM) for 5 min and stimulated with LPS (100 ng/mL) for 6 h. iNOS mRNA expression was quantified by real-time RT-PCR. Values are expressed as means ± SD of the percentage of iNOS mRNA expression compared with LPS alone from three independent experiments using different cell preparations. ***p<0.001, significantly different from basal. ###*p*<0.001, significantly different from LPS alone (ANOVA with Bonferroni’s post-test).

We then examined whether poly(P) affects the expression of iNOS mRNA. As shown in Figure 5, poly(P)_130_ (100 µM) treatment led to a significant decrease in iNOS mRNA expression induced by LPS, suggesting that poly(P) reduces LPS-induced iNOS expression at least in part by down-regulating the expression level of iNOS mRNA.

### Effects of poly(P) on LPS-induced TNF release

**Figure 6:**
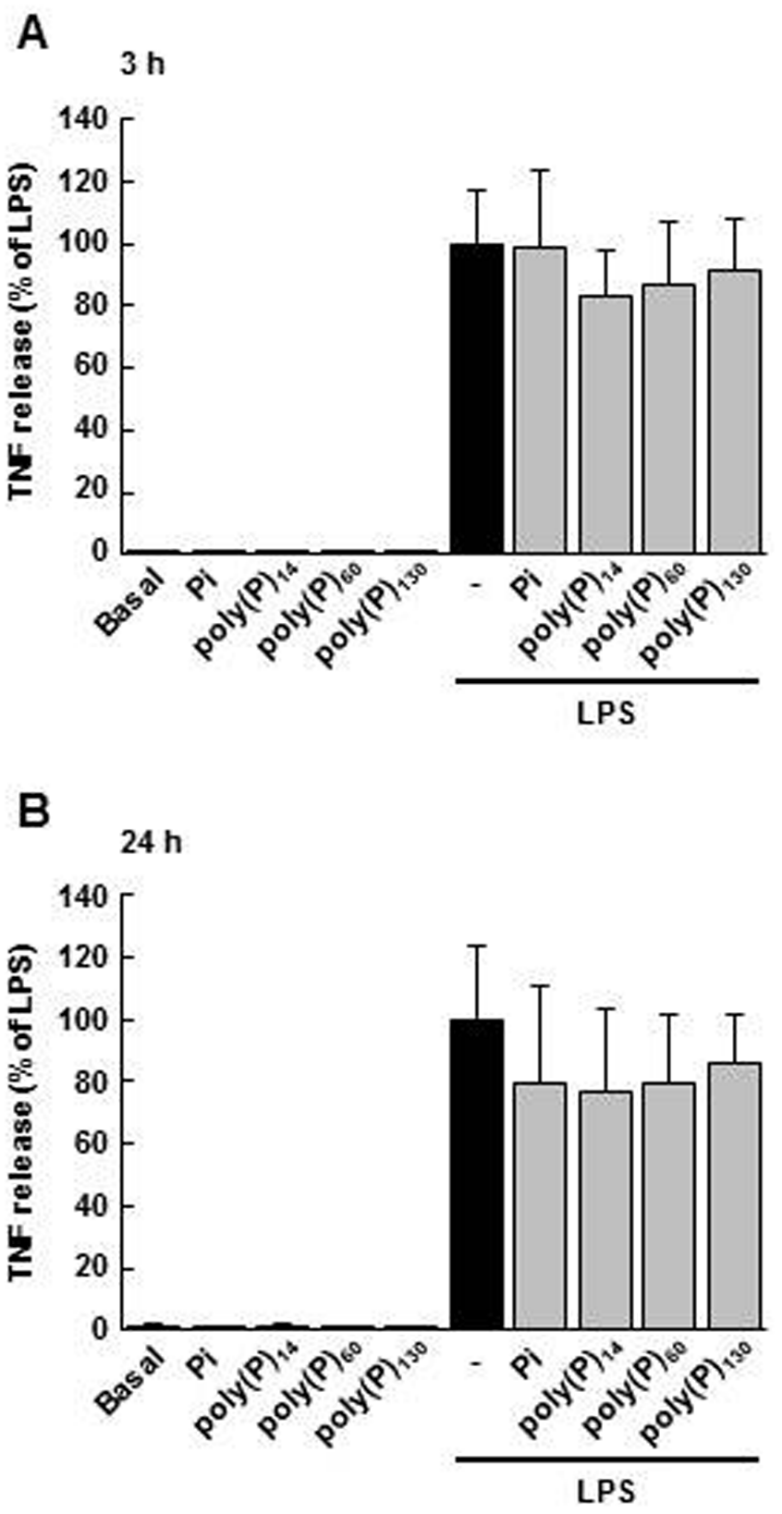
Effects of poly(P) on LPS-induced TNF release. The cells were pretreated with Pi, poly(P)_14,_ poly(P)_60_, or poly(P)_130_ (1 mM) for 5 min and stimulated with LPS (100 ng/mL) for 3 h (A) or 24 h (B). The released TNF was measured by ELISA. Values are expressed as means ± SD of the percentage of TNF release compared with LPS alone from three independent experiments using different cell preparations.

In addition to iNOS, the activation of TLR4 by LPS causes the expression of various inflammatory mediators including TNF in macrophages [20]. Thus, we examined whether poly(P) could suppress TNF release from LPS-stimulated macrophages as well as iNOS expression. For the TNF assay, macrophages were pretereated with poly(P)_14_, poly(P)_60_, or poly(P)_130_ for 5 min, and then stimulated with LPS, similar to iNOS. After the 3-h or 24-h stimulation with LPS, the amount of TNF released was significantly elevated. However, poly(P)_14_, poly(P)_60_, and poly(P)_130_ did not have significant effects on LPS-induced TNF release (Figure 6A and B). Moreover, the 24-h pretreatment with poly(P) also did not suppress LPS (3 h)-induced TNF release (the percentage of TNF release compared with LPS: LPS + poly(P)_14_, 101.4%±13.3%; LPS + poly(P)_60_, 98.0%±17.1%; LPS + poly(P)_130_, 101.7%±10.4%; mean ± SD; n  =  3). Therefore, it seems that poly(P) does not inhibit all the macrophage responses to LPS.

## Discussion

In mammalian cells, poly(P) is ubiquitously present; it seems to exert various functions. Previous studies have shown that poly(P) is involved in blood coagulation [5]–, the function of channels [10], [11], cell apoptosis [12], activation of mTOR [13], energy metabolism [14], and the differentiation and calcification of osteoblasts [3], [4]. In this study, we further showed that extracellular inorganic poly(P) reduces iNOS expression induced by LPS stimulation in mouse peritoneal macrophages.

LPS is a cell wall component of Gram-negative bacteria and potently activates TLR4 thus inducing the expression of various inflammation-associated genes including iNOS. NO produced in macrophages in response to infection plays an important role in early defense responses of the innate immunity by killing pathogens. However, large amounts of NO may also induce the death of host cells. Thus, NO production must be tightly regulated. Our results indicate that poly(P) suppresses LPS-induced iNOS expression in macrophages. Therefore, poly(P) can serve as a negative modulator of NO production in response to infection. In addition, a recent study reported that poly(P) suppressed the induction of proinflammatory cytokines IL-1β and IL-6 in an experimental model of acute colitis [27]. Thus, poly(P) may modulate the production of these cytokines as well as NO upon infectious diseases.

In mammals, various lengths of poly(P) seem to be present in different tissues and cells. Reportedly, poly(P) observed in rodent heart, kidneys, lungs, liver and brain is 50–800 phosphate residues long [15]. In contrast, only relatively short chain lengths of poly(P), composed of 60–100 phosphate residues, accumulate in the granules of platelets and mast cells [7], [17], [26]. We showed that poly(P) exerts a suppressive effect on iNOS expression at lengths of 14 to 130 phosphate residues. Thus, it is possible that endogenous poly(P) suppresses LPS-induced iNOS expression. Microbial poly(P), which is hundreds to thousands of phosphate residues long, may also demonstrate this effect.

Endogenous poly(P) may serve as an intercellular signaling molecule in innate immunity. For example, platelets and mast cells store poly(P) in their granules and secrete it into the extracellular space when the cells are activated [7], [17], [26]. These poly(P)-accumulating granules are similar to acidocalcisomes, which are acidic organelles containing calcium and poly(P) in prokaryotes and lower eukaryotes, and such organelles are likely to be present in other types of mammalian cells [28]. Thus, such packed poly(P) may be released into the extracellular space and affect the function of macrophages, thereby serving as an immune modulator. Further, as poly(P) seems to be ubiquitously present, it may be leaked from injured cells when the immune reaction is overwhelming. Such poly(P) may reflect the degree of tissue damage and regulate NO production to avoid further damage expansion. On the other hand, pathogens may actively secrete their own poly(P), which may help them to escape from NO-producing macrophages by inhibiting iNOS expression.

Although our results suggest that poly(P) suppresses LPS-induced iNOS expression by regulating the level of mRNA expression, detailed mechanisms remain yet to be elucidated. It has been reported that LPS-induced extracellular Ca^2+^ influx enhances iNOS expression in macrophages [29], [30]. Poly(P), a negatively charged polyanion, may bind with extracellular Ca^2+^, inhibit LPS-induced Ca^2+^ influx, and result in a decreased expression of iNOS mRNA. On the other hand, extracellular poly(P) has been recently reported to modulate or activate receptors on the plasma membrane, such as the FGF, integrin, and P2Y_1_ receptors [2], [27], [31]. Thus, poly(P) may regulate iNOS expression via such receptors. In addition, considering that TNF release was not affected by poly(P), poly(P) may modulate iNOS expression via a signaling pathway that is not involved in TNF production.

In conclusion, our results indicate that extracellular inorganic poly(P) can decrease LPS-induced iNOS expression in mouse peritoneal macrophages. This novel finding suggests a link between poly(P) and innate immunity.

## References

[1] Rao NN, Gómez-García MR, Kornberg A (2009) Inorganic polyphosphate: essential for growth and survival. Annu Rev Biochem 78: 605–647. 19344251 10.1146/annurev.biochem.77.083007.093039

[2] Shiba T, Nishimura D, Kawazoe Y, Onodera Y, Tsutsumi K, et al (2003) Modulation of mitogenic activity of fibroblast growth factors by inorganic polyphosphate. J Biol Chem 278: 26788–26792. 12740373 10.1074/jbc.M303468200

[3] Kawazoe Y, Shiba T, Nakamura R, Mizuno A, Tsutsumi K, et al (2004) Induction of calcification in MC3T3-E1 cells by inorganic polyphosphate. J Dent Res 83: 613–618. 15271969 10.1177/154405910408300806

[4] Kawazoe Y, Katoh S, Onodera Y, Kohgo T, Shindoh M, et al (2008) Activation of the FGF signaling pathway and subsequent induction of mesenchymal stem cell differentiation by inorganic polyphosphate. Int J Biol Sci 4: 37–47. 18274622 10.7150/ijbs.4.37 PMC2238184

[5] Smith SA, Mutch NJ, Baskar D, Rohloff P, Docampo R, et al (2006) Polyphosphate modulates blood coagulation and fibrinolysis. Proc Natl Acad Sci U S A 103: 903–908. 16410357 10.1073/pnas.0507195103 PMC1347979

[6] Smith SA, Morrissey JH (2008) Polyphosphate enhances fibrin clot structure. Blood 112: 2810–2816. 18544683 10.1182/blood-2008-03-145755 PMC2556616

[7] Müller F, Mutch NJ, Schenk WA, Smith SA, Esterl L, et al (2009) Platelet polyphosphates are proinflammatory and procoagulant mediators in vivo. Cell 139: 1143–1156. 20005807 10.1016/j.cell.2009.11.001 PMC2796262

[8] Mutch NJ, Engel R, Uitte de Willige S, Philippou H, Ariëns RA (2010) Polyphosphate modifies the fibrin network and down-regulates fibrinolysis by attenuating binding of tPA and plasminogen to fibrin. Blood 115: 3980–3988. 20228273 10.1182/blood-2009-11-254029

[9] Choi SH, Smith SA, Morrissey JH (2011) Polyphosphate is a cofactor for the activation of factor XI by thrombin. Blood 118: 6963–6970. 21976677 10.1182/blood-2011-07-368811 PMC3245215

[10] Kim D, Cavanaugh EJ (2007) Requirement of a soluble intracellular factor for activation of transient receptor potential A1 by pungent chemicals: role of inorganic polyphosphates. J Neurosci 27: 6500–6509. 17567811 10.1523/JNEUROSCI.0623-07.2007 PMC6672444

[11] Zakharian E, Thyagarajan B, French RJ, Pavlov E, Rohacs T (2009) Inorganic polyphosphate modulates TRPM8 channels. PLoS One 4: e5404. 19404398 10.1371/journal.pone.0005404 PMC2671608

[12] Hernandez-Ruiz L, González-García I, Castro C, Brieva JA, Ruiz FA (2006) Inorganic polyphosphate and specific induction of apoptosis in human plasma cells. Haematologica 91: 1180–1186. 16956816

[13] Wang L, Fraley CD, Faridi J, Kornberg A, Roth RA (2003) Inorganic polyphosphate stimulates mammalian TOR, a kinase involved in the proliferation of mammary cancer cells. Proc Natl Acad Sci U S A 100: 11249–11254. 12970465 10.1073/pnas.1534805100 PMC208743

[14] Pavlov E, Aschar-Sobbi R, Campanella M, Turner RJ, Gómez-García MR, et al (2010) Inorganic polyphosphate and energy metabolism in mammalian cells. J Biol Chem 285: 9420–9428. 20124409 10.1074/jbc.M109.013011 PMC2843191

[15] Kumble KD, Kornberg A (1995) Inorganic polyphosphate in mammalian cells and tissues. J Biol Chem 270: 5818–5822. 7890711 10.1074/jbc.270.11.5818

[16] Leyhausen G, Lorenz B, Zhu H, Geurtsen W, Bohnensack R, et al (1998) Inorganic polyphosphate in human osteoblast-like cells. J Bone Miner Res 13: 803–812. 9610744 10.1359/jbmr.1998.13.5.803

[17] Moreno-Sanchez D, Hernandez-Ruiz L, Ruiz FA, Docampo R (2012) Polyphosphate is a novel pro-inflammatory regulator of mast cells and is located in acidocalcisomes. J Biol Chem 287: 28435–28444. 22761438 10.1074/jbc.M112.385823 PMC3436523

[18] Kawai T, Akira S (2011) Toll-like receptors and their crosstalk with other innate receptors in infection and immunity. Immunity 34: 637–650. 21616434 10.1016/j.immuni.2011.05.006

[19] Kumar H, Kawai T, Akira S (2011) Pathogen recognition by the innate immune system. Int Rev Immunol 30: 16–34. 21235323 10.3109/08830185.2010.529976

[20] Kawai T, Akira S (2010) The role of pattern-recognition receptors in innate immunity: update on Toll-like receptors. Nat Immunol 11: 373–384. 20404851 10.1038/ni.1863

[21] Wink DA, Hines HB, Cheng RY, Switzer CH, Flores-Santana W, et al (2011) Nitric oxide and redox mechanisms in the immune response. . J Leukoc Biol. 89: 873–891. 21233414 10.1189/jlb.1010550 PMC3100761

[22] Pautz A, Art J, Hahn S, Nowag S, Voss C, et al (2010) Regulation of the expression of inducible nitric oxide synthase. Nitric Oxide 23: 75–93. 20438856 10.1016/j.niox.2010.04.007

[23] Shiba T, Shiba T, Yamaoka M, Uematsu T, Takahashi Y, et al.. (2003) Polyphosphate-water soluble collagen complexes and process for preparation thereof. Japanese Patent Number 4698932.

[24] Shiba T, Takahashi Y, Uematsu T, Kawazoe Y, Ooi K, et al (2004) Effect of inorganic polyphosphate on periodontal regeneration. Key Engineering Materials 254-2: 1119–1122.

[25] Chen PS, Toribara TY, Warner H (1956) Microdetermination of phosphorus. Anal Chem 28: 1756–1758.

[26] Ruiz FA, Lea CR, Oldfield E, Docampo R (2004) Human platelet dense granules contain polyphosphate and are similar to acidocalcisomes of bacteria and unicellular eukaryotes. J Biol Chem 279: 44250–44257. 15308650 10.1074/jbc.M406261200

[27] Segawa S, Fujiya M, Konishi H, Ueno N, Kobayashi N, et al (2011) Probiotic-derived polyphosphate enhances the epithelial barrier function and maintains intestinal homeostasis through integrin-p38 MAPK pathway. PLoS One 6: e23278. 21858054 10.1371/journal.pone.0023278 PMC3156119

[28] Docampo R, Moreno SN (2011) Acidocalcisomes. Cell Calcium 50: 113–119. 21752464 10.1016/j.ceca.2011.05.012 PMC3156361

[29] Kim Y, Moon JS, Lee KS, Park SY, Cheong J, et al (2004) Ca^2+^/calmodulin-dependent protein phosphatase calcineurin mediates the expression of iNOS through IKK and NF-κB activity in LPS-stimulated mouse peritoneal macrophages and RAW 264.7 cells. Biochem Biophys Res Commun 314: 695–703. 14741691 10.1016/j.bbrc.2003.12.153

[30] Zhou X, Yang W, Li J (2006) Ca2+- and protein kinase C-dependent signaling pathway for nuclear factor-kappaB activation, inducible nitric-oxide synthase expression, and tumor necrosis factor-alpha production in lipopolysaccharide-stimulated rat peritoneal macrophages. J Biol Chem 281: 31337–31347. 16923814 10.1074/jbc.M602739200

[31] Holmström KM, Marina N, Baev AY, Wood NW, Gourine AV, et al (2013) Signalling properties of inorganic polyphosphate in the mammalian brain. Nat Commun 4: 1362. 23322050 10.1038/ncomms2364 PMC3562455

